# The clinical efficacy of Afatinib 30 mg daily as starting dose may not be inferior to Afatinib 40 mg daily in patients with stage IV lung Adenocarcinoma harboring exon 19 or exon 21 mutations

**DOI:** 10.1186/s40360-017-0190-1

**Published:** 2017-12-13

**Authors:** Chih-Jen Yang, Ming-Ju Tsai, Jen-Yu Hung, Mei-Hsuan Lee, Ying-Ming Tsai, Yu-Chen Tsai, Jui-Feng Hsu, Ta-Chih Liu, Ming-Shyan Huang, Inn-Wen Chong

**Affiliations:** 1 Department of Internal Medicine, Kaohsiung Municipal Ta-Tung Hospital, Kaohsiung Medical University, Kaohsiung, Taiwan; 2 Division of Pulmonary and Critical Care Medicine, Department of Internal Medicine, Kaohsiung Medical University Hospital, Kaohsiung Medical University, No. 100, Tzyou First Road, Kaohsiung City, Taiwan; 3 Division of Hematology and Oncology, Department of Internal Medicine, Kaohsiung Medical University Hospital, Kaohsiung Medical University, No. 100, Tzyou First Road, Kaohsiung City, Taiwan; 4 Faculty of Medicine, College of Medicine, Kaohsiung Medical University, Kaohsiung, Taiwan; 5 Graduate Institute of Clinical Medicine, College of Medicine, Kaohsiung Medical University, Kaohsiung, Taiwan; 6 Graduate Institute of Medicine, College of Medicine, Kaohsiung Medical University, Kaohsiung, Taiwan; 7 Department of Respiratory Therapy, College of Medicine, Kaohsiung Medical University, Kaohsiung, Taiwan

**Keywords:** Lung cancer, Adenocarcinoma, Afatinib, Epidermal growth factor receptor, Tyrosine kinase inhibitor;diarrhea, Adverse drug reaction

## Abstract

**Background:**

Afatinib is a second-generation epidermal growth factor receptor (EGFR) tyrosine kinase inhibitor (TKI). Compared to cytotoxic chemotherapy, afatinib has been shown to have better efficacy in the treatment of non-small cell lung cancer harboring *EGFR* mutations. However, 40 mg daily as the initial dose is often accompanied by serious adverse drug reactions (ADRs) and 28 to 53.3% of patients required a dose reduction. No previous study has compared the clinical efficacy and ADRs of different initial doses (40 mg vs. 30 mg daily) of afatinib in lung cancer treatment.

**Methods:**

Patients with stage IV lung adenocarcinoma diagnosed and treated in two Kaohsiung Medical University-affiliated hospitals in Taiwan between May 2014 and August 2016 were identified and followed until December 2016. Demographic characteristics, responses, progression-free survival (PFS), overall survival (OS), and ADRs were recorded.

**Result:**

A total of 48 patients with stage IV lung adenocarcinoma harboring susceptible *EGFR* mutations who received afatinib as their first-line therapy were enrolled. Patients using 30 mg daily as the initial dose tended to be older and female and have a smaller body size. The patients using 30 mg of afatinib daily as their initial dose had a similar response rate to those receiving 40 mg daily (76% vs. 95%, *p* = 0.0862) and the same disease control rate (100% vs. 100%, *p* = 0.1486). The PFS was similar between the patients receiving 30 mg or 40 mg of afatinib daily (median PFS: 469 vs. 443 days, log-rank *p* = 0.8418). Patients receiving 30 mg daily had a significantly lower incidence of diarrhea than those using 40 mg daily (41% vs. 100%, *p* < 0.0001).

**Conclusion:**

An initial afatinib dose of 30 mg daily had similar response and progression-free survival rates as an initial dose of 40 mg daily, but resulted in fewer serious ADRs in this study.

## Background

Lung cancer continues to be the leading cause of death among patients with malignant tumors worldwide. Large-scale studies have shown the efficacy of epidermal growth factor receptor (EGFR) tyrosine kinase inhibitors (TKIs) such as erlotinib, gefitinib, and afatinib in patients with non-small cell lung cancer (NSCLC) with susceptible *EGFR* mutations, including an increased tumor response rate and prolonged progression-free survival compared to cytotoxic chemotherapy as the first-line therapy [1–6].

Afatinib is an orally administered irreversible inhibitor of the ErbB family of tyrosine kinases, and it is regarded to be a second-generation *EGFR* TKI [7]. Compared to cytotoxic chemotherapy, afatinib has been shown to significantly prolong progression-free survival (PFS) but not overall survival (OS) in the overall population of patients harboring *EGFR* mutations and receiving chemotherapy as first-line therapy [1, 8]. However, afatinib has been shown to prolong both PFS and OS in patients with advanced lung adenocarcinoma and exon 19 deletions [9]. The LUX-Lung 7 trial reported that afatinib used as first-line treatment for patients with advanced lung adenocarcinoma and activating *EGFR* mutations significantly prolonged PFS and the time to treatment failure but not overall survival compared to gefitinib treatment [10].

Previously published clinical trials have used a standard initial dose of 40 mg daily of afatinib, however they have also reported high rates of severe adverse drug reactions (ADRs) including grade 3–4 diarrhea, skin rash, and paronychia [1, 8, 10]. In clinical practice, moderate-to-severe ADRs often lead to discontinuation of treatment or a dose reduction, while some patients even refuse to receive re-challenge.

Dose reductions in 53.3% (122/229) and 28.0% (67/239) of the patients using 40 mg of afatinib daily were reported in the LUX-Lung 3 and LUX-Lung 6 trials, respectively, with most of these reductions occurring within the first 6 months of treatment. Reducing the dose to 30 mg daily decreased the incidence of ADRs with a similar median PFS in subgroup analyses of the LUX-Lung 3 and LUX-Lung 6 trials [1, 8, 9, 11]. However, no previous study has compared the treatment efficacy of a different initial dose (30 mg or 40 mg daily) of afatinib as the first-line therapy in patients with lung adenocarcinoma harboring susceptible *EGFR* mutations.

In Taiwan, the National Health Insurance Bureau has permitted the use of both 30 mg and 40 mg daily of afatinib as the first-line therapy for patients with advanced lung adenocarcinoma with activating *EGFR* mutations since May 2014. Therefore, in this descriptive observational study, we reviewed all patients with lung adenocarcinoma harboring susceptible *EGFR* mutations who received a different initial dose of afatinib as the first-line *EGFR* TKI in two hospitals, and analyzed the clinical efficacy and ADRs to demonstrate the real world data in Taiwan.

## Methods

### Patient identification

Patients with stage IV lung adenocarcinoma diagnosed and treated between May 2014 and August 2016 in two Kaohsiung Medical University-affiliated hospitals (Kaohsiung Medical University Hospital and Kaohsiung Municipal Ta-Tung Hospital) in Taiwan were identified and followed until December 2016. The diagnosis of lung cancer was confirmed pathologically according to World Health Organization pathology classification, and tumor staging was made by a special committee including clinical pulmonologists, medical oncologists, chest surgeons, radiologists, pathologists and radiation oncologists, according to the seventh American Joint Committee on Cancer staging system. Patients were included if they: (1) had adequate tumor specimens for *EGFR* mutation examinations and had susceptible *EGFR* mutations including exon 19 deletions and exon 21 L858R point mutations; (2) were chemotherapy-naïve and treated with 30 mg or 40 mg daily of afatinib as the first-line treatment.

Baseline clinical characteristics were determined by retrospective chart review, including age at diagnosis, sex, Eastern Cooperative Oncology Group (ECOG) performance status at the beginning of first-line afatinib treatment, smoking history, and tumor histology. Glomerular filtration rate was estimated using the Modification of Diet in Renal Disease formula (eGFR-MDRD). Mutations in the *EGFR* gene were analyzed using an *EGFR* RGQ kit (Qiagen, UK) which utilized amplification refractory mutation specific (ARMS) polymerase chain reactions and Scorpion technology for detection and/or direct sequencing as in our previous report [12–16]. The initial treatment response was classified based on serial imaging studies using the revised Response Evaluation Criteria in Solid Tumors (RECIST 1.1) criteria. The PFS and OS with first-line treatment were defined as the duration from the start of the first treatment to the date of disease progression on imaging studies and the date of death, respectively. ADRs were recorded by physicians and graded according to the Common Terminology Criteria for Adverse Events (CTCAE) v4.0.

### Ethical consideration

The Institutional Review Board (IRB) of Kaohsiung Medical University Hospital (KMUH) approved this study (KMUHIRB-E(II)-20150162) and waived the need for written informed consent from all patients. In addition, patient records were anonymized and de-identified prior to analysis.

### Statistical analysis

Categorical variables and continuous variables were compared using the χ^2^ test and the Student’s t-test, respectively. Survival times were estimated using the Kaplan-Meier method, with differences between groups compared using the log-rank test. Using the backward variable selection method keeping only variables with *p* values less than 0.2, we developed reduced multivariable models with Cox regression analysis to determine the predictive factors for PFS and OS. Hazard ratios (HRs) with 95% confidence intervals (CIs) of the predictive factors were presented. All statistical analyses were performed using SAS software (version 9.4 for Windows, SAS Institute Inc., Cary, NC, USA). Statistical significance was set at a two-sided *p* value of less than 0.05.

## Results

### Patient characteristics

During the study period, 48 patients with stage IV lung adenocarcinoma harboring susceptible *EGFR* mutations who received afatinib as the first-line therapy were enrolled (Table 1), of whom 29 (60.5%) and 19 (39.5%) received 30 mg and 40 mg daily as their initial treatment, respectively. The patients who received 30 mg daily as the initial dose tended to be older (67.3 ± 8.0 vs. 60.6 ± 8.8 years, *p* = 0.0090) and female (79% vs. 37%) with a lower weight (55.4 ± 9.3 vs. 62.2 ± 6.8 kg, *p* = 0.0091) and lower height (156.8 ± 7.6 vs. 162.0 ± 7.6 cm, *p* = 0.0055) compared to those who received 40 mg daily. There were no significant differences in smoking history, performance status, number of metastatic sites on initial diagnosis, eGFR-MDRD, serum albumin level, thyroid transcription factor-1 or EGFR gene mutations (exon 19 or 21) between the two groups. The median (interquartile range) of the observation period was 334.5 (218.5–456) days [322 (231–421) and 361 (197–506) days for the patients initially using 30 mg and 40 mg of afatinib daily, respectively].

**Table 1:** Clinical characteristics and treatment response of all patients

Variables	All patients	Afatinib 30 mg daily	Afatinib 40 mg daily	*P* value
*N* (%)	48	29	19	
Age (year) -mean ± SD	64.6 ± 8.9	67.3 ± 8.0	60.6 ± 8.8	0.0090
Age -*n* (%)				0.0280
< 65 years old	26 (54%)	12 (41%)	14 (74%)	
≥ 65 years old	22 (46%)	17 (59%)	5 (26%)	
Sex -n (%)				0.0030
Female	30 (63%)	23 (79%)	7 (37%)	
Male	18 (38%)	6 (21%)	12 (63%)	
Smoking history -*n* (%)				0.0509
Never smoker	43 (90%)	28 (97%)	15 (79%)	
Ever smoker	5 (10%)	1 (3%)	4 (21%)	
TTF-1 staining -*n* (%)				
Positive	47 (98%)	29 (100%)	18 (95%)	
Not performed	1 (2%)	0 (0%)	1 (5%)	
EGFR gene mutation site -*n* (%)				0.3720
Exon19	29 (60%)	19 (66%)	10 (53%)	
Exon21	19 (40%)	10 (34%)	9 (47%)	
Performance status while starting afatinib -*n* (%)				0.4861
ECOG ≤1	38 (79%)	22 (76%)	16 (84%)	
ECOG ≥2	10 (21%)	7 (24%)	3 (16%)	
Number of metastatic sites on initial diagnosis -*n* (%)				0.1689
≤ 1 site	21 (44%)	15 (52%)	6 (32%)	
≥ 2 sites	27 (56%)	14 (48%)	13 (68%)	
Metastatic sites on initial diagnosis -*n* (%)				
Brain metastasis	12 (25%)	8 (28%)	4 (21%)	0.6092
Lung metastasis	14 (29%)	6 (21%)	8 (42%)	0.1104
Pleural metastasis/pleural effusion	21 (44%)	12 (41%)	9 (47%)	0.6825
Bone metastasis	28 (58%)	17 (59%)	11 (58%)	0.9602
Liver metastasis	2 (4%)	1 (3%)	1 (5%)	0.7583
Adrenal metastasis	7 (15%)	3 (10%)	4 (21%)	0.3040
Weight (kg) -mean ± SD	58.1 ± 9	55.4 ± 9.3	62.2 ± 6.8	0.0091
Height (cm) -mean ± SD	158.9 ± 7.9	156.8 ± 7.6	162.0 ± 7.6	0.0240
Body surface area (m^2^) -mean ± SD	1.6 ± 0.2	1.5 ± 0.2	1.7 ± 0.1	0.0055
Body mass index (kg/m^2^) -mean ± SD	23.0 ± 2.9	22.5 ± 3.4	23.7 ± 1.9	0.1332
Serum creatinine level (mg/dL) -mean ± SD	1.1 ± 1.9	1.0 ± 1.7	1.3 ± 2.2	0.6464
eCCr-CG (mL/min) -mean ± SD	74.6 ± 28.4	69.8 ± 25.3	82.1 ± 31.7	0.1439
eGFR-MDRD (mL/min) -mean ± SD	89.9 ± 31.4	89.5 ± 29.7	90.5 ± 34.6	0.9151
Serum albumin (mg/dL)	3.9 ± 0.6	3.8 ± 0.6	4.1 ± 0.5	0.0757

*Abbreviations: eCCr-CG estimated creatinine clearance rate using Cockcroft-Gault formula, *eGFR-MDRD* estimated glomerular filtration rate using Modification of Diet in Renal Disease formula

### Similar outcomes with either 30 mg or 40 mg daily of afatinib as the first-line treatment

**Fig. 1:**
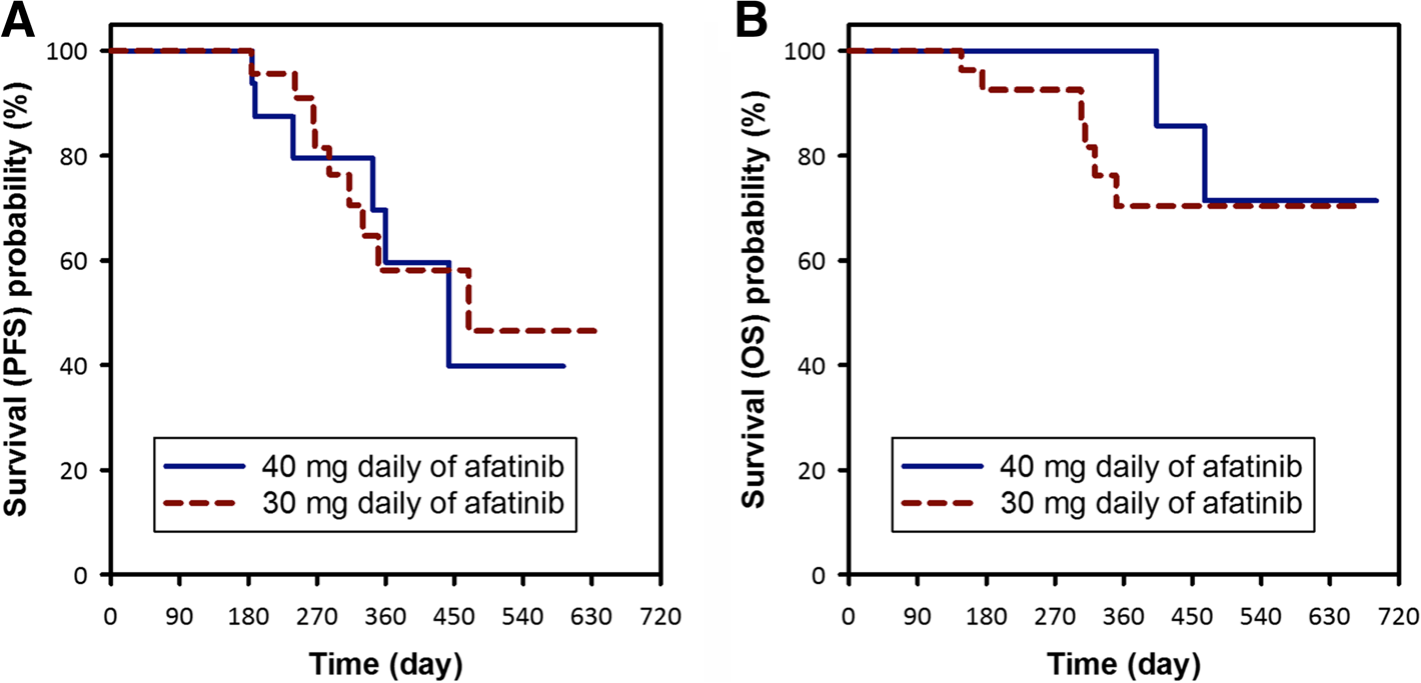
The progression-free survival (PFS) (**a**) and overall survival (OS) (**b**) were similar between the patients receiving 30 mg and 40 mg daily of afatinib (median PFS: 469 vs. 443 days, log-rank *p* = 0.8418; log-rank *p* = 0.3522 for OS)

The patients who received an initial dose of 30 mg daily of afatinib had a similar response rate (76% vs. 95%, *p* = 0.0862) and the same disease control rate (100% vs. 100%) as those who received an initial dose of 40 mg daily (Table 2). The PFS was also similar between the two groups (median PFS: 469 (30 mg) vs. 443 days (40 mg), log-rank *p* = 0.8418) (Fig. 1a), and there as no significant difference in OS (log-rank *p* = 0.3522) (Fig. 1b). To identify the factors predicting treatment outcomes, we used backward variable selection with the variables afatinib dose, age, sex, smoking history, EGFR mutation site, number of metastatic sites, weight, height, eGFR-MDRD, and serum albumin level in multivariable Cox regression models (Table 3). The independent predictive factors for PFS were male sex (HR [95% CI] = 4.45 [1.14–17.42], *p* = 0.0322) and number of metastatic sites (HR [95% CI] = 8.40 [1.90–37.23], *p* = 0.0051). The performance status when starting afatinib was the only independent predictive factor for OS (HR [95% CI] = 6.01 [1.30–28.21], *p* = 0.0219).

**Table 2:** Initial treatment response to different initial afatinib doses

Variables	All patients	Afatinib 30 mg daily	Afatinib 40 mg daily	*P* value
Initial response to afatinib treatment -*n* (%)				0.1486
Complete response	1 (2%)	1 (3%)	0 (0%)	
Partial response	39 (81%)	21 (72%)	18 (95%)	
Stable disease	8 (17%)	7 (24%)	1 (5%)	
Disease control rate with afatinib treatment (%)	48 (100%)	29 (100%)	19 (100%)	0.1486
Response rate with afatinib treatment (%)	40 (83%)	22 (76%)	18 (95%)	0.0862

**Table 3:** Multivariate Cox regression analysis to identify the outcome predictors

Variables	Hazard ratio [95% CI]	*P* value
Predictive factors for progression-free survival (PFS)		
Afatinib dose (40 mg daily vs. 30 mg daily)	0.40 [0.11–1.49]	0.1722
Sex (male vs. female)	4.45 [1.14–17.42]	0.0322
Smoking history (ever smokers vs. never smokers)	0.19 [0.02–2.24]	0.1867
Number of metastatic sites (≥2 sites vs. ≤1 site)	8.40 [1.90–37.23]	0.0051
Predictive factors for overall survival (OS)		
Performance status while starting afatinib (ECOG ≥2 vs. ECOG ≤1)	6.01 [1.30–28.21]	0.0219

*The candidate variables included for selection were afatinib dose, age, sex, smoking history, EGFR mutation site of the cancer specimen, number of metastatic sites, weight, height, estimated glomerular filtration rate using the Modification of Diet in Renal Disease formula, and serum albumin level. Multivariable Cox regression models were built using the backward variable selection method, keeping only variables with *p* values less than 0.2, to determine the predictive factors for PFS and OS

### Adverse drug reactions of the patients using 30 mg or 40 mg daily of afatinib as the first-line treatment

The most common ADRs in the patients taking afatinib included skin rash and/or acneiform eruptions (85%), dry skin (71%), and diarrhea (65%) (Table 4). The patients who received 30 mg daily of afatinib had a significantly lower incidence of diarrhea than those receiving 40 mg daily (41% vs. 100%, *p* < 0.0001). A grade 3 skin rash occurred in three patients (16%) in the 40 mg group compared to no patients in the 30 mg group. However, because of the limited number of cases, we could not make a definitive conclusion about the development of a skin rash between the two groups. Finally, four patients (21%) in the 40 mg group had a reduction in dose to 30 mg daily due to ADRs; the median interval for a dose reduction was 120 days. No patients discontinued treatment or reduced the dose in the 30 mg group.

**Table 4:** Adverse events related to afatinib

Adverse events	All patients	Afatinib 30 mg daily	Afatinib 40 mg daily	*P* value
Presence of adverse events				
Diarrhea	31 (65%)	12 (41%)	19 (100%)	<0.0001
Moderate-to-severe (≥ Grade 2)	3 (6%)	1 (3%)	2 (11%)	0.3218
Stomatitis	10 (21%)	5 (17%)	5 (26%)	0.4490
Moderate-to-severe (≥ Grade 2)	2 (4%)	0 (0%)	2 (11%)	0.0743
Paronychia	24 (50%)	14 (48%)	10 (53%)	0.7679
Moderate-to-severe (≥ Grade 2)	13 (27%)	9 (31%)	4 (21%)	0.4466
Skin rash and/or acneiform eruption	41 (85%)	25 (86%)	16 (84%)	0.8480
Moderate-to-severe (≥ Grade 2)	8 (17%)	4 (14%)	4 (21%)	0.5093
Dry skin	34 (71%)	20 (69%)	14 (74%)	0.7250
Moderate-to-severe (≥ Grade 2)	1 (2%)	0 (0%)	1 (5%)	0.2118
Pruritus	12 (25%)	6 (21%)	6 (32%)	0.3942
Moderate-to-severe (≥ Grade 2)	0 (0%)	0 (0%)	0 (0%)	
Details of adverse events				
Diarrhea				0.0004
Grade 0	17 (35%)	17 (59%)	0 (0%)	
Grade 1	28 (58%)	11 (38%)	17 (89%)	
Grade 2	2 (4%)	1 (3%)	1 (5%)	
Grade 3	1 (2%)	0 (0%)	1 (5%)	
Stomatitis				0.2033
Grade 0	38 (79%)	24 (83%)	14 (74%)	
Grade 1	8 (17%)	5 (17%)	3 (16%)	
Grade 2	2 (4%)	0 (0%)	2 (11%)	
Paronychia				0.1013
Grade 0	24 (50%)	15 (52%)	9 (47%)	
Grade 1	11 (23%)	5 (17%)	6 (32%)	
Grade 2	9 (19%)	8 (28%)	1 (5%)	
Grade 3	4 (8%)	1 (3%)	3 (16%)	
Skin rash and/or acneiform eruption				0.1354
Grade 0	7 (15%)	4 (14%)	3 (16%)	
Grade 1	33 (69%)	21 (72%)	12 (63%)	
Grade 2	5 (10%)	4 (14%)	1 (5%)	
Grade 3	3 (6%)	0 (0%)	3 (16%)	
Dry skin				0.4461
Grade 0	14 (29%)	9 (31%)	5 (26%)	
Grade 1	33 (69%)	20 (69%)	13 (68%)	
Grade 2	1 (2%)	0 (0%)	1 (5%)	
Pruritus				0.3942
Grade 0	36 (75%)	23 (79%)	13 (68%)	
Grade 1	12 (25%)	6 (21%)	6 (32%)	

* Data are presented as n(%)

## Discussion

In patients with susceptible *EGFR* mutations receiving treatment with an *EGFR* TKI for NSCLC, discontinuing treatment or reducing the dose is not uncommon because of intolerable or high-grade ADRs [1, 8, 9, 11, 17]. The clinical efficacy of a lower dose of gefitinib or erlotinib has been shown to be non-inferior to a standard dose of gefitinib for NSCLC with susceptible *EGFR* mutations [18–21]. However, no previous study has compared the treatment efficacy of a standard dose and lower dose of afatinib. To the best of our knowledge, this study is the first to demonstrate similar response rates, PFS, and OS between the use of 30 mg or 40 mg afatinib daily as the initial treatment for stage IV lung adenocarcinoma with susceptible *EGFR* mutations. The incidence of diarrhea was significantly lower in the 30 mg group than in the 40 mg group, and up to 21% of the patients in the 40 mg group had a reduction in dose to 30 mg due to ADRs.

Afatinib is a second generation *EGFR* TKI, which irreversibly inhibits the ErbB family of tyrosine kinases. In a pooled analysis of the LUX-Lung 3 and LUX-Lung 6 trials, afatinib showed a statistically significant PFS benefit compared to chemotherapy (HR [95% CI]: 0.42 [0.34–0.53] [15]. The pooled analysis also showed a significant improvement in OS in the subgroup of patients with cancer cells harboring exon 19 deletions [9, 15].

The median PFS rate among patients harboring exon 19 and exon 21 mutations and receiving 40 mg afatinib as the initial dose was 10.9 to 13.6 months in the LUX-Lung trials [1, 8–10]. In our study, the median PFS rates in the 30 mg and 40 mg groups were 15.6 months and 14.8 months, respectively. Despite this improvement in PFS with *EGFR* TKI treatment, almost no *EGFR-*TKI-based trials have shown a benefit in OS compared to cytotoxic chemotherapy. In the current study, male sex and multiple metastatic sites were both independent predictive factors for PFS, however the initial treatment dose was not. In addition, the performance status when starting afatinib was the only independent predictive factor for OS in this study. However, since the median OS was not reached in either group because most patients were still alive in December 2016, studies with a longer follow-up period are warranted to confirm our preliminary findings.

In the LUX-Lung 7 trial, a phase 2B, open-label randomized controlled trial enrolling patients with NSCLC harboring susceptible *EGFR* mutations, the patients who received afatinib had a significantly longer PFS (median: 11.0 vs. 10.9 months, *p* = 0.017) and time-to-treatment failure (median: 13.7 vs. 11.5 months, *p* = 0.0073) than those who received gefitinib as the initial *EGFR* TKI [10]. However, serious ADRs were reported in 11% of the patients taking 40 mg of afatinib daily compared to 4% of those taking gefitinib, and the dose was reduced in 42 and 2% of the patients taking afatinib and gefitinib, respectively [10]. In the LUX-Lung 3 and LUX-Lung 6 trials, around 28 to 53.3% of the patients had the dose of afatinib reduced [1, 8, 9, 11].

Furthermore, in pooled analysis of the LUX-Lung 3 and LUX-Lung 6 trials, the most common afatinib-related grade 3–4 ADRs were rash or acne (15–16%), diarrhea (5–14%), paronychia (11%), and stomatitis or mucositis (5%) [1, 8, 9, 11]. In addition, a recent large meta-analysis of 2535 patients who received first- and second-generation *EGFR* TKIs reported that about 40% of the patients experienced grade 3–4 ADRs, and that the risk for grade 3–4 ADRs was significantly lower in the patients taking gefitinib (29.1%) than in those receiving erlotinib (54.1%) or afatinib (42.1%) (*p* < 0.01) [22]. Takeda et al. also reported discontinuation of treatment due to ADRs in 7.7% of patients [17].

Taken together, these findings imply that although afatinib has good treatment efficacy, the ADRs resulting from 40 mg daily are a serious consideration. In the current study, up to 21% of the patients initially taking afatinib 40 mg daily had to reduce the dose to 30 mg daily due to ADRs. In contrast, none of patients receiving afatinib 30 mg daily reduced their dose or discontinued treatment.

Skin rash and diarrhea are the most frequent ADRs in patients receiving *EGFR* TKIs, and several meta-analyses have reported a significantly higher risk of skin rash with afatinib (84.8%) than with erlotinib (62.0%) or gefitinib (62.0%) (*p* < 0.01) [17, 22]. In the current study, 16% of the patients in the 40 mg group had a grade 3 skin rash compared to none of the patients in the 30 mg group, suggesting that a lower dose of afatinib might carry a lower risk of severe skin rash. In addition, in the aforementioned meta-analyses, the risk of diarrhea was significantly higher with afatinib (91.7%) than with erlotinib (42.4%) or gefitinib (44.4%) (*p* < 0.01) [17, 22]. In the current study, all of the patients in the 40 mg group had diarrhea, although only two patients (11%) had moderate-to-severe diarrhea. In contrast, only 41% of patients in the 30 mg group had diarrhea, and only one patient (3%) had moderate-to-severe diarrhea.

There are several limitations to this study. First, the number of cases was relatively small, and this was a descriptive observation study rather than a retrospective analytic study. Afatinib has been covered by the Taiwan National Health Insurance program since May 2014, which is why the number of patients who received afatinib is much lower than for those who received gefitinib or erlotinib. Second, we excluded some patients who had rare mutations such as exon 18 mutations or exon 20 mutations or insertions. These rare mutations almost always suggest poor efficacy, and therefore we excluded these patients to decrease intra-group and inter-group heterogeneity. Third, the follow-up time was too short to calculate median OS, since more than half of the patients were still alive at the end of December 2016. Further studies with a longer follow-up period are needed to confirm our preliminary findings with regards to OS. Fourth, we did not check serum concentrations of afatinib to monitor the pharmacokinetic profiles in patients taking different doses of afatinib. Whether a higher dose of afatinib would result in a higher serum level remains to be elucidated.

## Conclusion

We demonstrated that in patients with stage IV lung adenocarcinoma harboring susceptible *EGFR* mutations, an initial dose of 30 mg daily of afatinib may not be inferior to 40 mg daily with regards to response rate, PFS, and OS. In addition, diarrhea was significantly more common in the 40 mg daily group, and up to 21% of the patients who received afatinib 40 mg daily had to reduce the dose due to ADRs. Further prospective studies should be designed to compare the treatment efficacy, ADRs, and serum concentration between patients receiving different initial doses of afatinib.

## Abbreviations

EGFR: Epidermal growth factor receptor
OS: Overall survival
PFS: Progression free survival
TKIs: Tyrosine kinase inhibitors
